# An Unusual Tumor: Squamous Cell Carcinoma of the Colon in Lynch Syndrome

**DOI:** 10.7759/cureus.64477

**Published:** 2024-07-13

**Authors:** Aaron S Sidhu, Harneet Grewal

**Affiliations:** 1 Internal Medicine, Abrazo Community Health Network, Glendale, USA

**Keywords:** right hemicolectomy, microcytic anemia, (crc) colorectal carcinoma, lynch syndrome phenotype, scc-squamous cell carcinoma

## Abstract

Primary squamous cell carcinoma of the colon and rectum is a rare malignancy. Most of the anatomical sites that are reported to be affected include the esophagus and anal canal. This report highlights the case of a 54-year-old male with a known history of Lynch syndrome and a previous diagnosis of colon cancer who was found to have a recurrence of malignancy affecting this unlikely area. The treatment strategies for this colorectal squamous cell carcinoma have not been thoroughly explored, so this report aims to highlight effective interventions, including surgical resection and neoadjuvant chemotherapy and radiation. There is a poor prognosis associated with this condition, as it does not typically present until the late stages; however, in this particular instance, early detection leads to improved outcomes.

## Introduction

Primary squamous cell carcinomas of the colon and rectum are extremely rare, accounting for less than 1% of all colorectal malignancies [1] with the majority being related to adenocarcinoma [2]. They usually present in the fifth decade of life with a slight prevalence in men. Typically, squamous cell carcinomas are seen in the esophagus or anal canal [3]; however, this case focuses on the involvement in the colon. It has been discovered that 5-10% of all cases of colonic carcinomas can be hereditary, with the most common hereditary etiology being due to Lynch syndrome. Colorectal cancers associated with Lynch syndrome exhibit defective mismatch repair (MMR), resulting in microsatellite instability (MSI) and a high mutation burden [4]. The etiology, pathogenesis, and optimal treatment strategies for colorectal squamous cell carcinoma are not well established, so this report highlights a case of squamous cell carcinoma (SCC) affecting the colon in a patient with a history of Lynch syndrome to provide a brief overview of this rare condition. These findings typically present in an emergency setting, but in this case, the patient had a more indolent course with close follow-up that was not discovered until the patient experienced a progressive decline in status.

## Case presentation

**Figure 1:**
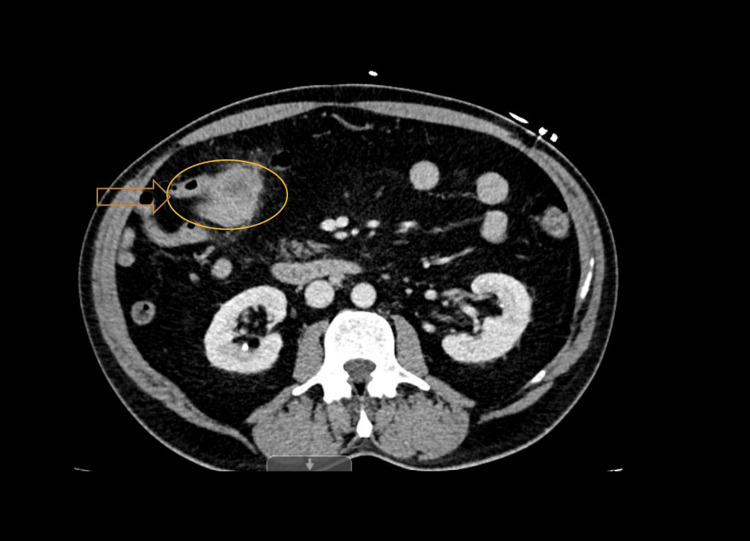
Abdominal CT scan post-contrast showing a 4.9 cm apple-core-like mass at the right-sided ileocolic anastomotic area concerning for primary or recurrent colon cancer

**Figure 2:**
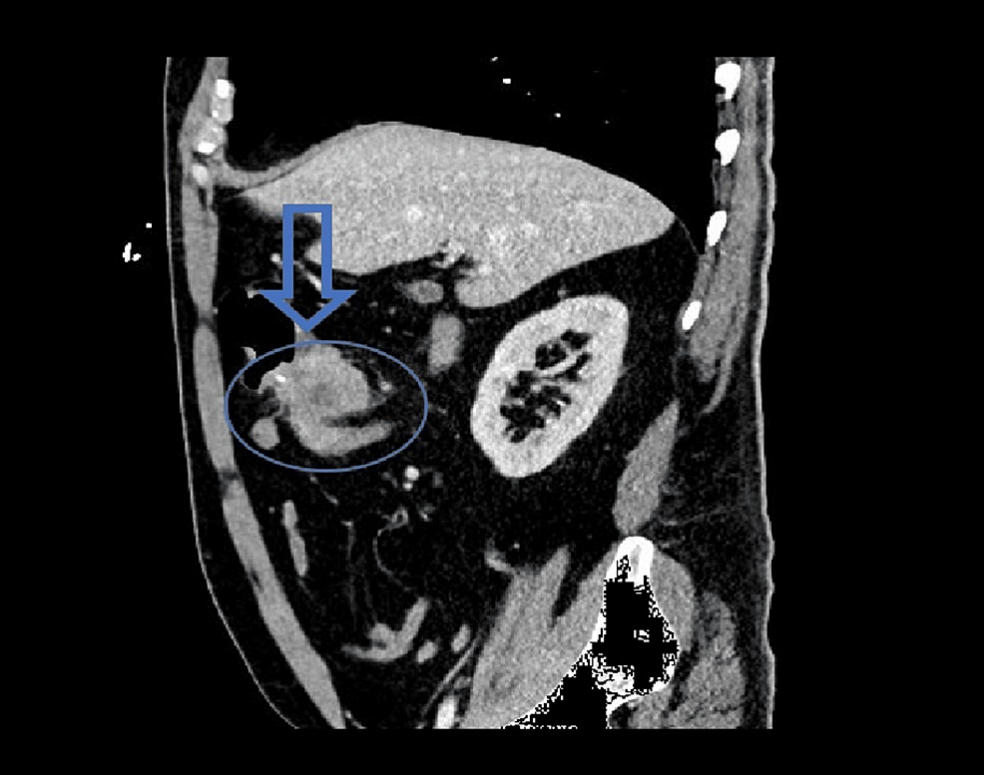
Sagittal CT scan post-contrast revealing an irregular mass at the ileocolic anastomosis site with enlarged mesenteric lymph nodes

This is a case of a 54-year-old male who presented to the emergency department with symptoms of lightheadedness, dizziness, and a recent history of dark and bloody stools. The patient had a known history of Lynch syndrome since adolescence and was subsequently diagnosed with colon cancer at age 40. He had previously undergone a right-sided hemicolectomy approximately 15 years ago upon this initial diagnosis. Six months before this most recent admission, he was noted to be profoundly anemic and underwent endoscopy and colonoscopy during that time with no significant findings. On initial evaluation, he was found to have severe microcytic anemia despite administrating multiple IV iron infusions throughout the last few months. A CT scan of the abdomen and pelvis noted a 4.9 cm apple-core-like mass at the right-sided ileocolic anastomotic area concerning for primary or recurrent colon cancer (Figure 1). Enlarged mesenteric lymph nodes within the right side, consistent with regional metastatic disease, were also noted (Figure 2). A repeat upper endoscopy was remarkable for Grade B erosive esophagitis, but no peptic ulceration or active bleeding was seen. A repeat colonoscopy revealed a 1.5-2cm ulcerated and friable polypoid lesion at the level of the ileocolonic anastomosis, and a biopsy was obtained. Pathology results from the biopsy revealed moderately differentiated squamous cell carcinoma at the anastomosis site of the colon, with no evidence of lymph node involvement or metastasis. The histology and immunostains supported the diagnosis of squamous cell carcinoma, however, they were negative for colon primary. Using TNM (tumor, node, metastasis) criteria, the patient was determined to have T2N0M0 staging given the submucosal involvement with no spread to lymph nodes or distant sites. Colorectal surgery explained that due to this reoccurrence of malignancy, surgical resection would be necessary. In addition, given the patient's history of Lynch syndrome, he was referred for oncological follow-up, but chemotherapy was deferred pending the surgical resection. The patient did not have any further evidence of overt bleeding and was stable enough to be discharged with specific diet restrictions, with instructions to follow up if he continued to have significant bleeding episodes.

## Discussion

Squamous colorectal carcinoma, despite being rare and accounting for 0.1-0.25% of all colorectal malignancies, can be detected in any part of the colon. There have been about 150 documented cases of colorectal SCC in the last 100 years, with the majority being discovered in the rectum and the second most frequent being the right colon. Furthermore, in association with Lynch syndrome, there has been only one previously reported case of squamous cell colorectal carcinomas. Lynch syndrome is responsible for 2% to 4% of all colorectal cancer cases and is associated with an up to 80% increase in the lifetime risk of colorectal cancer [1]. Screening for Lynch syndrome involves criteria such as Amsterdam II and Bethesda, which rely heavily on family history and clinical presentation but have low sensitivity. Pathological analysis via immunohistochemistry (IHC) and MSI testing is necessary for confirmation. In addition, it generally presents at a later stage and is associated with a poor prognosis. It is essential to obtain multiple biopsies from the suspected lesion during colonoscopy, as SCC can present anywhere, from a polyp to a malignant-appearing lesion [5]. It Is also important to remember to exclude the presence of SCC elsewhere in the body before a diagnosis of primary SCC is made. Surgical resection is the gold standard of treatment, however, neoadjuvant chemotherapy and radiotherapy have developed a principal role recently [6]. The evidence supporting the use of immunotherapy in mismatch repair deficient (dMMR) tumors, including those associated with Lynch syndrome, is indeed rapidly growing. This has culminated in significant advancements, including the first Food and Drug Administration (FDA) approval for molecular-guided, tissue-agnostic immunotherapy. Checkpoint inhibitors, such as PD-1 inhibitors like pembrolizumab and nivolumab, have shown remarkable efficacy in these tumors [3]. Given the high risk of developing multiple cancers in patients with Lynch syndrome and the effectiveness of immunotherapy in dMMR tumors, adopting a molecular-driven approach to management could lead to more personalized and effective treatments [4]. As patients with Lynch syndrome live longer with early detection and treatment of their cancers, unusual sites and histology of previously unreported cancers may emerge, as is illustrated by this case. This case aims to increase awareness of primary colorectal SCC to clinicians, specifically in association with Lynch syndrome, particularly due to the poor prognosis associated with late-stage presentation.

## Conclusions

This presents a case of primary squamous cell carcinoma of the colon and rectum in a 54-year-old man. SCC of the colon and rectum is exceedingly rare, and the clinical manifestations, treatment, and prognosis remain poorly defined. Many studies suggest that surgical resection or neoadjuvant chemotherapy and radiation have a beneficial role in most of these cases, and this has been the gold standard of treatment in such cases. In conjunction with Lynch syndrome, which has been determined to be the most common hereditary cause of SCC in the colon and rectum, genetic testing has been instrumental in advancements in diagnosis and treatment. There has been an increasing role for immunotherapy in these mismatch repair deficient tumors, leading to FDA approval for molecular-guided therapies that are personalized to the individual. With improvements in early detection and treatment for Lynch syndrome-associated malignancies, these studies will be critical in improving long-term outcomes for patients who were previously believed to have a poor prognosis.

## References

[1] Ali Husain AH, Kaundinya KB, Hammed F, Al Sayed AR (2020). The surprise pathology—primary squamous cell carcinoma of the colon—a case report. Int J Surg Case Rep.

[2] Gelas T, Peyrat P, Francois Y (2002). Primary squamous-cell carcinoma of the rectum: report of six cases and review of the literature. Dis Colon Rectum.

[3] Juturi JV, Francis B, Koontz PW, Wilkes JD (1999). Squamous-cell carcinoma of the colon responsive to combination chemotherapy. Report of two cases and review of the literature. Dis Colon Rectum.

[4] Miyamoto H, Nishioka M, Kurita N (2007). Squamous cell carcinoma of the descending colon: report of a case and literature review. Case Rep Gastroenterol.

[5] Novak ER, Wooddruff JD (1963). Gynaecologic and Obstetric Pathology: With Clinical and Endocrine Relations—Fifth Edition. Calif Med.

[6] Vasens H, Watson P, Mecklin JP, Lynch H (1999). New clinical criteria for hereditary nonpolyposis colorectal cancer (HNPCC, Lynch syndrome) proposed by the International Collaborative Group on HNPCC. Gastroenterology.

